# Performance analysis of a new hypersonic vitrector system

**DOI:** 10.1371/journal.pone.0178462

**Published:** 2017-06-06

**Authors:** Paulo Eduardo Stanga, Salvador Pastor-Idoate, Isaac Zambrano, Paul Carlin, David McLeod

**Affiliations:** 1 Manchester Royal Eye Hospital, Central Manchester University Hospitals NHS Foundation Trust, Manchester Academic Health Science Centre, Manchester, United Kingdom; 2 Manchester Vision Regeneration (MVR) Lab at Manchester Royal Eye Hospital and NIHR/ Wellcome Trust Manchester CRF, Manchester, United Kingdom; 3 Centre for Ophthalmology and Vision Sciences, Institute of Human Development, University of Manchester, Manchester, United Kingdom; 4 Eye Bank, Manchester Royal Eye Hospital, Manchester, United Kingdom; 5 Operating Theatre Services, Manchester Royal Eye Hospital, Manchester, United Kingdom; Massachusetts Eye & Ear Infirmary, Harvard Medical School, UNITED STATES

## Abstract

**Purpose:**

To evaluate porcine vitreous flow and water flow rates in a new prototype hypersonic vitrectomy system compared to currently available pneumatic guillotine vitrectors (GVs) systems.

**Methods:**

Two vitrectors were tested, a prototype, ultrasound-powered, hypersonic vitrector (HV) and a GV. Porcine vitreous was obtained within 12 to 24 h of sacrifice and kept at 4°C. A vial of vitreous or water was placed on a precision balance and its weight measured before and after the use of each vitrector. Test parameters included changes in aspiration levels, vitrector gauge, cut rates for GVs, % ultrasound (US) power for HVs, and port size for HVs. Data was analysed using linear regression and *t-*tests.

**Results:**

There was no difference in the total average mean water flow between the 25-gauge GV and the 25-gauge HV (*t*-test: *P* = 0.363); however, 25-gauge GV was superior (*t*-test: *P* < 0.001) in vitreous flow. The 23-gauge GV was only more efficient in water and vitreous removal than 23-gauge HV needle-1 (Port 0.0055) (*t*-test: *P* < 0.001). For HV, wall thickness and gauge had no effect on flow rates. Water and vitreous flows showed a direct correlation with increasing aspiration levels and % US power (p<0.05).

**Conclusions:**

The HV produced consistent water and vitreous flow rates across the range of US power and aspiration levels tested. Hypersonic vitrectomy may be a promising new alternative to the currently available guillotine-based technologies.

## Introduction

Significant reductions in the gauge of pneumatic guillotine vitrectors (GVs) have decreased ocular trauma [1–5]; resulting in quicker recovery and less postoperative discomfort for patients [1, 5–9]. However, concomitant reductions in port or lumen size of the GVs are accompanied by reductions in vitreous flow through the vitrector needle [10–11].

Efforts to overcome this reduction in flow volume have been directed toward reducing the viscosity of the vitreous by increasing the cut rate of GVs [12–13]. However, there is a mechanical speed limit created by the speed of the vitreous cutter blade and the cutter duty cycle. These factors condition the maximum cut rate [1–2, 5, 11, 14].

There are other limitations related to GVs.

First, turbulence is created by the periodic opening-and closing of the port. Increasing the cut rate partially reduces this turbulence, but any cutter with a periodically closed port will produce this effect.

Second, vitreous material may be caught between the inner needle and the port edges. Rather than being cut, the vitreous is aspirated uncut, or partially cut, through the port, resulting in direct traction on the vitreous strands.

Third, as the cutting action requires the viscous material to be drawn into the port past the outer needle before it can be cut, there is a natural limit to how close a surgeon can bring the active cutting region to the retina.

Fourth, the outer needle port must be large enough to permit a reasonable amount of tissue to enter to achieve a cut.

These issues, combined with interest in ever-smaller-gauge instruments, require higher infusion pressures to support flow through the larger port when the needle is not cutting and overcome the resistance of the smaller diameter lumen [9, 11].

The hypersonic vitrectomy system uses low amplitude ultrasonic (US) motion of the tip to create oscillating high speed flows near the port that ‘cut’ vitreous. It also liquefies the vitreous in the vicinity of the tip to the viscosity of water. This allows the hypersonic vitrector (HV) to address some of the limitations of GVs. The US HV has a single needle instead of two needles, so there is no chance of trapping vitreous strands between the port edge and the needle. The port is continuously open, allowing smaller port sizes and larger inner-lumen diameters. This, in turn, lowers flow resistance and infusion pressures.

The purpose of this study was to evaluate the feasibility and efficacy of a prototype US-based HV and to compare fluidics outcomes with those of a currently available GV.

## Material and methods

All animal tissue samples were treated in accordance with applicable laws for research involving animal tissues and samples (MRC 2004-Biomedical Research) and in accordance with the Declaration of Helsinki. The study using animal cadaveric tissues and samples was approved by the Manchester Royal Eye Hospital Steering Committee (R03781, Nov 2014).

### Guillotine and hypersonic vitrectors

**Fig 1:**
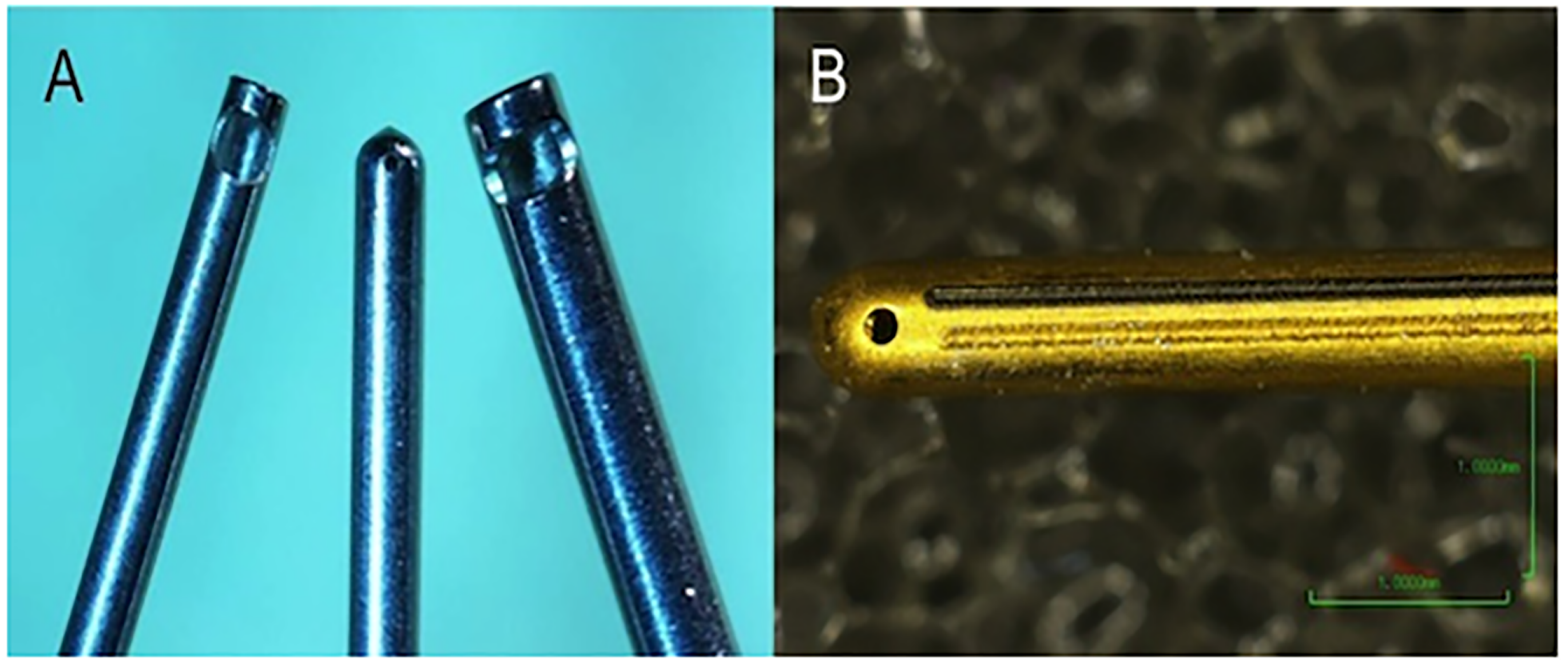
(**A)** 23-gauge HV needle (center) compared with 25-gauge (right) and 23-gauge (left) of guillotine needle. (**B)** High-magnification image of the tip and port of the HV.

The HV has an ultrasonically driven handpiece with a closed end needle and a small port located on the side at the end of the needle. The GV is a pneumatically driven handpiece with a closed end needle and a small port located on the end of the needle. The HV needle is about 33 mm long, similar to the needle length of 23 gage GV devices. In addition to the outer needle, the GV handpiece has a moving inner needle, typically about three gages smaller than the outer needle. The HV handpiece does not have a cut rate, but operates at a fixed ultrasound frequency around 28.5 kHz, and a peak-to-peak amplitude between 10 μm and 50 μm. (Fig 1).

GV gut rates are determined by the drive frequency, and were driven at up to 5000 CPM (0.086 kHz) in this study; the inner needle typically moves approximately 1 mm (1000 um) inside the nominally stationary outer needle. Wall thickness and port size of the HV needle were varied as part of the test series; port lengths in the GV are 0.019” to 0.021”.

### Test parameters

Water and vitreous flow rates for all vitrector needle configurations were tested on a StellarisPC with reduced BL3270 handpiece drive levels to run the HV tips, using the Venturi vacuum pump to provide aspiration vacuum. Test conditions included aspiration levels from 50–600 mmHg, cut rates (GV) from 0–5000 cuts per minute (CPM), US power (HV) from 0–50%, gauge (23-gauge and 25-gauge), wall thickness (0.003–0.005 in) for the 23-gauge HV and port size (0.0055–0.012 in) for the 23-gauge HV (Rochester, NY).

### Measuring water and vitreous flow rates

Each GV and HV needle was suspended above a vial of water or vitreous placed on a high-sampling (2 samples per second), precision (0.0001 g) balance (Adam Equipment PW 254 balance, Oxford CT, USA) that measured the mass of water or vitreous. Six 30 second measurements were taken manually, 3 before and 3 after each trial, by two observers and results were converted to volume removed as a function of time (ml/min), using the arithmetic mean of weight calculations of the two observers. All vitrectors were held vertically with a spring-clamp. Porcine vitreous was harvested en bloc within 12–24 h of local slaughter (Nixon’s Farm Shop Cheadle SK8 3PS, Manchester, UK) and kept at 4°C. One sample was taken for each combination of parameters; almost 1000 data points were taken in all.

### Statistical analysis

The water and vitreous flow rate average, standard deviation, minimum and maximum values were calculated for each GV and HV gauge and aspiration level; GV CPM; and HV US power, wall thickness and port size. Student´s *t-test* was used to compare minimum and maximum cut rate. Repeated measures of analysis of variance tested mean water and vitreous flow rates across cut rates. Mixed models with repeated measures were used to obtain regression equations to predict mean water and vitreous flow rates. One-way ANOVA testing was used to compare mean water and vitreous flow rates by wall thickness and port size in the 23-gauge HV needle trials. Linear regression with a forced-zero intercept was used to identify the magnitude of flow dependencies on design parameters (Minitab Statistical Software, Minitab, State College, PA, USA; Microsoft Excel, Microsoft, Redford, WA, USA). The accepted level of significance for all tests was a *P* value < 0.05.

Where appropriate, cross-terms were included. For instance, where flow rate might be simultaneously influenced by vacuum, power and port area, the product of the three parameters was also included in a multiple regression. In these cases, a second regression against only those terms that were statistically significant by the *P* value was performed.

Input value parameters were normalized to reasonable parameter increments (cut rate 1000 CPM; power 10%; vacuum 100 mmHg; and port area proportional to the area of a 0.007 in port).

## Results

### Water flow for GVs

Water flow rates for 25- and 23-gauge GVs at various aspiration levels and cut speeds are depicted in S1A–S1D Fig. The results are summarized in Table 1. In general, water flow rates for both 25- and 23-gauge GVs increased with increasing aspiration levels and decreased with increasing cut rates; peaking at 0 CPM and decreasing as rates advanced to 5000 CPM. These trends were significant at all aspiration levels and CPM tested (*P* < 0.05). Analysis of variance of flow rates across cut rates was statistically significant (*P* < 0.001) at all aspiration levels and gauges.

**Table 1:** Multiple regression models using mixed repeated measures models: Predicting guillotine vitrector water and vitreous flow rates for increasing aspiration levels and cut rate.

	Parameter Estimate ± Standard Error		
	n	Mean water flow rates	*P* value
25-gauge			
R^2^ = 0.95			
Intercept = 0			
Aspiration (per 100mmHg Increase)	64	1.8 +/- 0.06 ml/min/100 mmHg	<0.001
Cuts/minute (per 1000 increase)	64	-0.45 +/- 0.07 ml/min /1000 CPM	<0.001
23-gauge			
R^2^ = 0.97			
Intercept = 0			
Aspiration (per 100mmHg Increase)	64	3.9 +/- 0.1 ml/min/100 mmHg	<0.001
Cuts/minute (per 1000 increase)	64	-1.0 +/- 0.1 ml/min /1000 CPM	<0.001
	n	Mean vitreous flow rates	*P* value
25-gauge			
R^2^ = 0.70			
Intercept = 0			
Aspiration (per 100mmHg Increase)	64	0.19 +/- 0.02 ml/min/100 mmHg	<0.001
Cuts/minute (per 1000 increase)	64	0.03 +/- 0.03 ml/min /1000 CPM	= 0,25
23-gauge*			
R^2^ = 0.74			
Intercept = 0			
Aspiration (per 100mmHg Increase)	63	0.23 +/- 0.03 ml/min/100 mmHg	<0.001
Cuts/minute (per 1000 increase)	63	0.07 +/- 0.03 ml/min /1000 CPM	= 0,03

* The 23-gauge data series was missing the 500 mmHg, 1500 CPM data point.

Water flow rates using the 23-gauge GV were 2.3 ± 0.23 times those of the 25-gauge GV at each CPM level and at each aspiration level at or above 200 mmHg (n = 40). Below 200 mmHg, the water flow rate was 1.6 ± 3.2 times that of the 25-gauge GV at 50 mmHg and 100 mmHg (n = 16).

### Vitreous flow for GVs

Vitreous flow rates for 25- and 23-gauge GVs at various aspiration levels and cut speeds are depicted in S2A–S2D Fig. The results are also summarized in Table 1. Vitreous flow rates for 25- and 23-gauge GVs increased with increasing aspiration levels. This trend was significant at all aspiration levels tested (*P* < 0.05). Vitreous flow rates increased with increasing CPM and peaked at 5000 CPM. Analysis of variance of flow rates across all aspiration levels was statistically significant (*P* < 0.05) at all aspiration rates, cut rates and gauges except the 25-gauge CPM relationship.

Vitreous flow rates using 23-gauge GVs were 1.88 ± 0.87 times those of the 25-gauge GV at each CPM level, and for aspiration levels at or above 200 mmHg (n = 39). Below 200 mmHg, the vitreous flow rate was 3.9 ± 4.0 times that of the 25-gauge GV for 50 mmHg and 100 mm Hg (n = 16).

### Water flow for HVs

Examples of water flow rates for 25- and 23-gauge HVs at various aspiration levels and US powers and port sizes are depicted in S3A–S3D Fig. The results of all measurements are summarized in Table 2. Wall thickness of HV needles had no impact on water flow rates (*P* < 0.730). Flow rates for 25-gauge HVs increased with increases in US power, although the trend was not significant (*P* = 0.41), and showed a significant trend for increased flow rate with increased aspiration levels (*P* < 0.001). Water flow rates for all port sizes increased with increasing aspiration levels (*P* < 0.001). In general, the contribution of power to water flow was either not statistically significant, or was small compared to the contribution of aspiration vacuum.

**Table 2:** Multiple linear regression models using mixed repeated measures models: Predicting hypersonic vitrector water flow rates for increasing aspiration and % US power.

				HV Water Flow Increase (ml/min), Zero Intercept Condition, Linear ANOVA fit, no cross-terms						
Needle	Port diameter (inches)	Wall thickness (inches)	n	ml /min per 10% power increment	*P* value	ml/min per 100 mmHg aspiration increment	*P* value	Linear fit adjusted R^2^	Standard error (ml/min)	F significance
25-gauge	0.0055	0.004	48	0.08 ± 0.093	0.41	1.22 ± 0.08	< 0.001	0.87	1.47	2.90E-23
23-gauge-1	0.0055	0.004	48	0.17 ± 0.039	< 0.001	1.27 ± 0.04	< 0.001	0.96	0.61	6.30E-41
23-gauge-2	0.007	0.003	48	-0.07 ± 0.15	0.68	2.63 ± 0.14	< 0.001	0.91	2.45	5.60E-27
23-gauge-3	0.009	0.004	48	0.31 ± 0.09	0.0013	3.68 ± 0.08	< 0.001	0.97	1.45	2.20E-44
23-gauge-4	0.010	0.004	48	0.42±0.12	0.001	4.2± 0.10	< 0.001	0.96	1.91	4.50E-42
23-gauge-5	0.012	0.004	48	0.96± 0.18	< 0.001	4.9 ± 0.16	< 0.001	0.96	2.8	1.00E-38
23-gauge-6	0.0055	0.003	48	0.09 ± 0.06	0.19	1.30 ± 0.06	< 0.001	0.93	1.05	1.20E-30
23-gauge-7	0.0055	0.005	48	0.15 ± 0.03	< 0.001	1.16 ± 0.03	< 0.001	0.96	0.52	2.40E-42

When port size and wall thickness were held constant (port size of 0.0055 in and wall thickness of 0.004 in), the 100 mm Hg increment in water flow rates using the 23-gauge HV (1.27 +/- 0.04 ml/min per 100 mm Hg) were not materially different from flow rates using the 25 gage HV (1.22 +/- 0.08 ml/min per 100 mm Hg). Thus, water flow through the HVs did not depend on gauge size.

### Modelling water flow for HVs

Opportunities for improving the data fit were considered. A model including two multi-parameter cross-terms (one for the product of port area and the square root of vacuum and a second for the product of area and vacuum (Flow = C1 * area * √vacuum + C2 * area * vacuum) was constructed. This model fit the observations with an adjusted R2 value of 0.967, (N = 348, F significance P = 2.8 * 10–290) which was highly statistically significant. Power was normalized to 10% steps, vacuum to 100 mmHg steps and area to the area of a 0.007 in diameter port (0.025 mm2). The derived coefficients were:

$$\begin{array}{l} C1=2.21\pm 0.18\;\mathrm{ml}/\min/[\left(\mathrm{area}/0.025\;\mathrm{mm}2\right)*\left(\surd \left(\mathrm{vacuum}/100\;\mathrm{mmHg}\right)\right]\;\left(P=4*10\text{-}29\right)\\ C2=1.033\pm 0.088\;\mathrm{ml}/\min/\left[\left(\mathrm{area}/0.025\;\mathrm{mm}2\right)*\left(100\;\mathrm{mmHg vacuum}\right)\right]\;\left(P=2*10\text{-}27\right) \end{array}$$

The standard error in the model was 1.9 ml/min of flow across the data set. With this cross-term included, the additional non-crossed linear relationships were not statistically significant:

$$\begin{array}{l} \begin{array}{l} \mathrm{Power}\;\left(0.1\;\mathrm{ml}/\min/10\%\;\mathrm{power},\;P=0.06\right)\\ \mathrm{Wall thickness}\;\left(\text{-}39\;\mathrm{ml}/\min/0.001\;\mathrm{in wall thickness},\;P=0.83\right)\\ \mathrm{Gauge}\;\left(\text{-}0.01\;\mathrm{ml}/\min/\mathrm{unit increment in gauge},\;P=0.70\right)\\ \mathrm{Vacuum}\;\left(0.022\;\mathrm{ml}/\min/100\;\mathrm{mmHg vacuum},\;P=0.10\right) \end{array}\\ \mathrm{Gauge size}\;\left(0.001\;\mathrm{ml}/\min/\mathrm{gauge value},\;P=0.30\right) \end{array}$$

Adding a linear term for vacuum provided a slightly different model with statistical significance for the coefficient (0.29 ml/min / 100 mmHg vacuum, P = 2*10–8), but with only a 0.2% increase in R2 and only a 5% decrease in the standard error. The additional complexity of the model did not make the model materially more meaningful.

The square root of vacuum term was introduced as the port acts much like a small orifice for water flow and flow is generally proportional to the square of the pressure difference across the orifice. Use of either the root vacuum*area cross-term or the vacuum*area cross-term by themselves resulted in an increase in standard error of about 25%.

**Fig 2:**
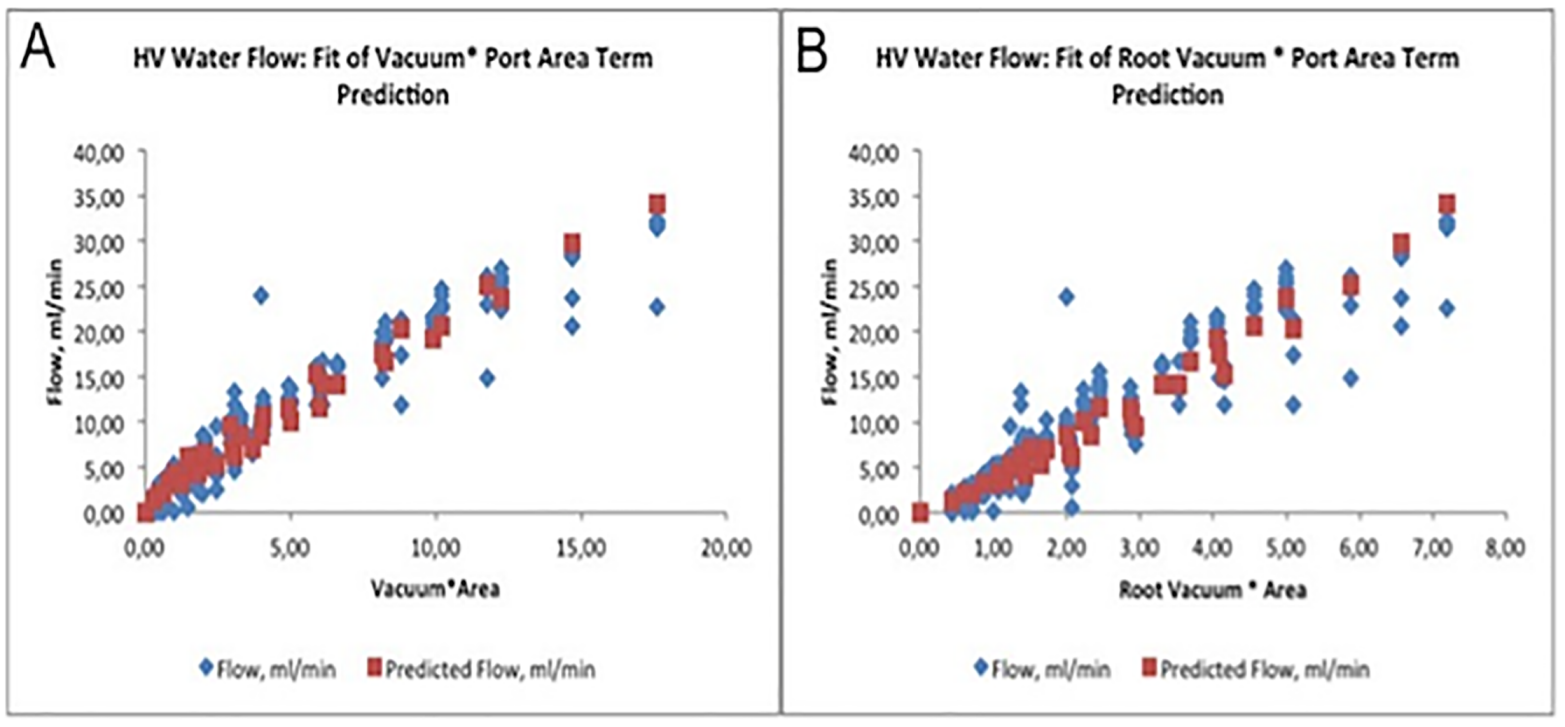
**(A)** Predicted and actual water flow through HV cutter for Vacuum * port area cross term. (**B)** Predicted and actual water flow through HV device for √Vacuum * port area cross term.

A line plot of the model fit to the data is presented in Fig 2A and 2B, along with a table identifying expected flow rates (Table 3).

**Table 3:** HV water flow (ml/min) as a function of port diameter and vacuum.

	Vacuum (mm Hg)					
Port diameter (in)	100	200	300	400	500	600
0.0055	2.5	4.1	5.4	6.7	7.9	9.1
0.007	3.2	5.2	6.9	8.6	10.1	11.6
0.009	4.2	6.7	8.9	11.0	13.0	14.9
0.010	4.6	7.4	9.9	12.2	14.4	16.6
0.012	5.6	8.9	11.9	14.7	17.3	19.9

### Vitreous flow for HVs

Vitreous flow rates for 25- and 23-gauge HVs at various US powers and aspiration levels are depicted in S4A and S4B Fig. The results are summarized in Table 4.

**Table 4:** Multiple regression models using mixed repeated measures models, predicting vitreous flow rates for increasing aspiration and % US power.

				HV cutter Vitreous Flow Increase, ml/min, Zero Intercept Condition, individual linear regression fit, no cross-terms						
Needle	Port diameter (inches)	Wall thickness (inches)	n	ml/min per 10% power increment	*P* value	ml /min per 100 mmHg vacuum increment	*P* value	Linear fit adjusted R^2^	Standard error (ml/min)	F significance
25-gauge	0.0055	0.004	48	0.035 ± 0.009	< 0.001	0.019 ± 0.008	0.029	0.51	0.15	2.00F-08
23-gauge—1	0.0055	0.004	48	0.060± 0.017	< 0.001	0.062 ± 0.015	< 0.001	0.62	0.27	4.00E-11
23-gauge—2	0.007	0.003	56	0.140 ± 0.033	< 0.001	0.17 ± 0.03	< 0.001	0.76	0.54	2.40E-18
23-gauge—3	0.009	0.004	56	0.210± 0.042	< 0.001	0.21 ± 0.038	< 0.001	0.77	0.69	4.80E-19
23-gauge—4	0.010	0.004	48	0.110 ± 0.031	< 0.001	0.23± 0.028	< 0.001	0.80	0.49	1.00E-17
23-gauge—6	0.0055	0.003	48	0.079 ± 0.012	< 0.001	0.029 ± 0.01	0.007	0.69	0.18	3.20E-13
23-gauge—7	0.0055	0.005	48	0.037± 0.012	0.004	0.050 ± 0.011	< 0.001	0.06	0.19	3.2E-11

No vitreous data was taken on the 23-gauge needle 5 (0.012” port, 0.004” wall)

Wall thickness of HVs needles had no impact on vitreous flow rates (*P* < 0.470). Vitreous flow rates for all port sizes (25- and 23-gauge) increased with increasing US power, a trend that was significant at all aspiration levels tested (*P* < 0.05).

### Modelling vitreous flow for HVs

Opportunities for improving the data fit were considered. A model including only a single multi-parameter cross-term for port area, power and vacuum (Flow = constant*area*vacuum*power) was constructed. This model fit the 352 observations with an adjusted R2 value of 0.828, (P value of 3.8 * 10–137) that was highly statistically significant. Power was normalized to 10% steps, vacuum to 100 mmHg steps and area to the area of a 0.007 in diameter port (0.025 mm2). The derived coefficient was:

C=0.0621±0.0015ml/min/[(10%power)*(100mmHgvacuum)*(0.025mm2portarea)]

The standard error in the cross-term model was 0.37 ml/min of flow across the data set. With this cross-term included, the additional non-crossed linear relationships to power (0.015 ml/min / 10% power, P = 0.30) vacuum (0.022 ml/min / 100 mmHg vacuum, P = 0.10) and gauge size (0.001 ml/min / gauge value, P = 0.30) were not statistically significant. Adding a linear term for port area provided a slightly different model, but reduced the R2 value from 0.82 to 0.76 while lowering the coefficient for the cross-term. This resulted in no change to the standard error and increased the P value for the cross-term to 1.0*10–95. The additional complexity of the model did not make the model more meaningful or improve the quality of the fit, and the simpler model was retained. Adding a term including the square root of the vacuum, as was added for the water flow model, did not improve the fit as it did in the water flow model.

**Fig 3:**
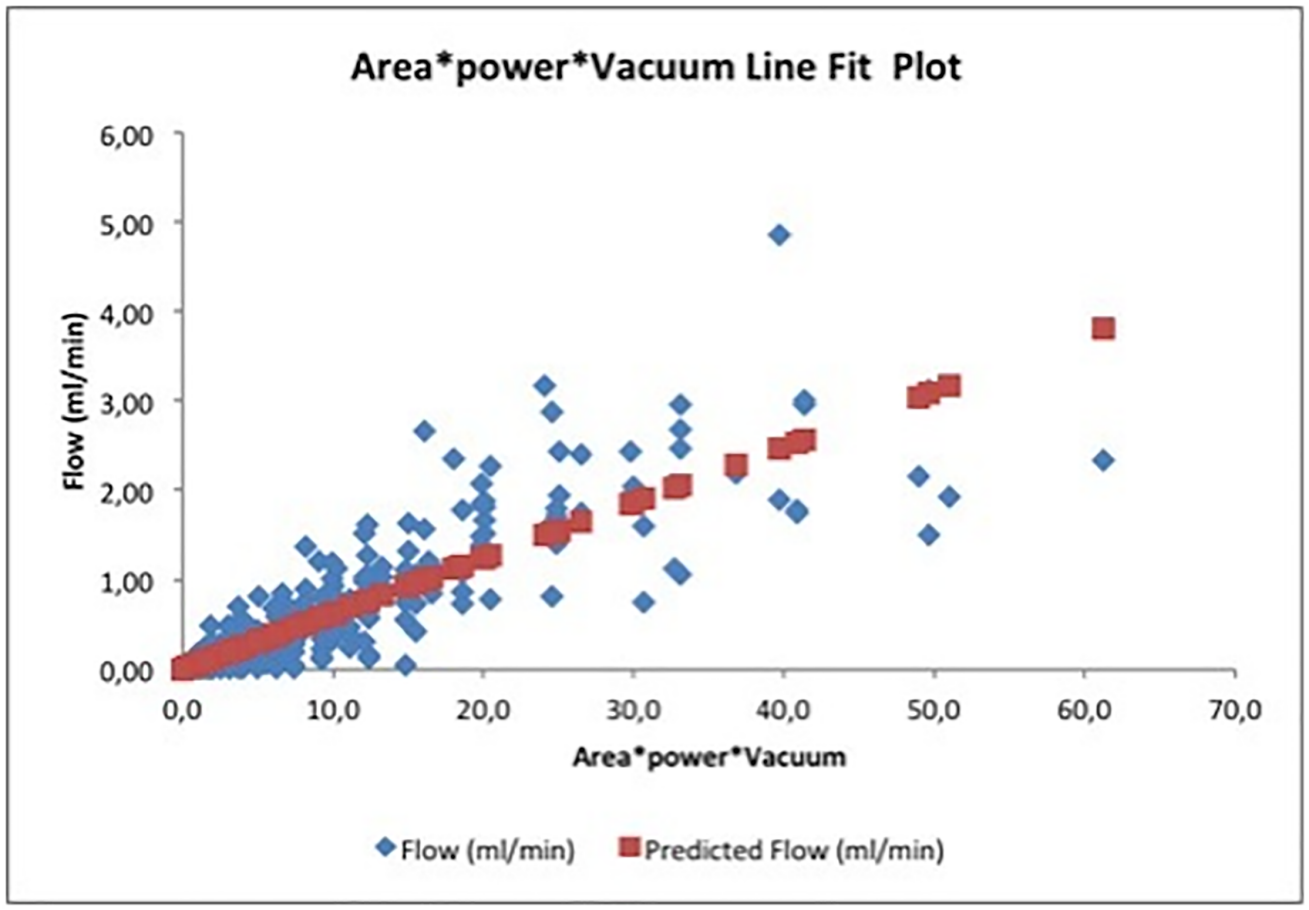
Predicted and actual vitreous flow through HV cutter for vacuum* port area cross term.

A line plot of the model fit to the data is included (Fig 3), along with two tables identifying aspiration and power sensitivity for the 0.007 in port HV (Table 5) and a table identifying expected flow rates (Table 6).

**Table 5:** Increase in vitreous flow rate by increments of power or vacuum.

Increase in vitreous flow (ml/min) through 0.007" diameter port per 10% increase in applied power at various vacuum levels (mm Hg)						
Vacuum, mm Hg:	100	200	300	400	500	600
Flow increase, ml/min / 10% power increment	0.062	0.124	0.186	0.249	0.311	0.373
Increase in vitreous flow (ml/min) through 0.007" diameter port per 100 mmHg increase in vacuum at various power levels (%)						
Power, %		10	20	30	40	50
Flow increase, ml/min / 100 mm Hg vacuum increment		0.062	0.124	0.186	0.249	0.311

**Table 6:** Typical vitreous flow (ml/min) through 0.007 in diameter port at various vacuums and powers.

	Vacuum (mmHg)					
US Power (%)	100	200	300	400	500	600
10	0.06	0.12	0.19	0.25	0.31	0.37
20	0.12	0.25	0.37	0.50	0.62	0.75
30	0.19	0.37	0.56	0.75	0.93	1.12
40	0.25	0.50	0.75	0.99	1.24	1.49
50	0.31	0.62	0.93	1.24	1.55	1.86

### Comparison of flow rates: GV vs. HV

#### Water flow

There was no difference in the total average mean water flow between the 25-gauge GV (4.09±3.60 ml/min) and the 25-gauge HV (3.49±2.90 ml/min) (*t*-test: *P* = 0.363). The average mean water flow rate for the 23-gauge GV (8.96±8.01 ml/min) was greater than that of the 23-gauge HV needle 1 (3.93 ± 2.54 ml/min) (*t*-test: *P* < 0.001).

In testing the effect of port size, there were no differences in water flow rate between the 23-gauge GV and the HV with needles 2 (7.28±3.97 ml/min) (*t*-test: P = 0.184) or 3 (11.0 ± 5.56 ml/min) (*t*-test: *P* = 0.122). The 23-gauge GV was had a lower flow rate in water than the HV with needles 4 (12.8±6.48 ml/min) (*t*-test: *P* = 0.006) or 5 (14.4±6.92 ml/min) (*t*-test: *P* < 0.001).

#### Vitreous flow

**Fig 4:**
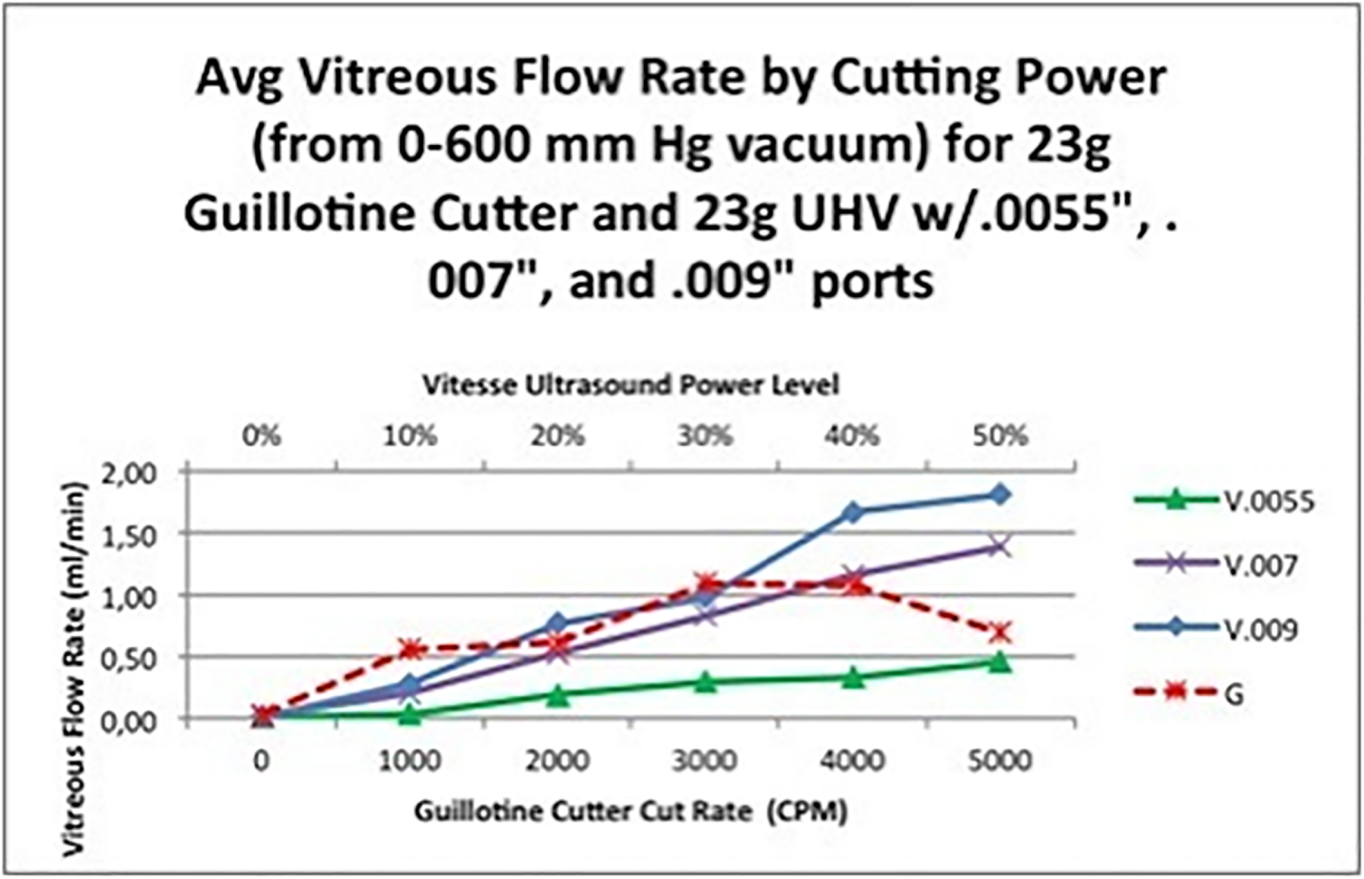
Average vitreous flow rate by cutting power for 23-gauge GV and 23-gauge HV cutters.

Total average mean vitreous flow with the 25-gauge GV (0.47±0.66 ml/min) was superior to the 25-gauge HV (0.11±0.18 ml/min) (*t*-test: *P* < 0.001). The 23-gauge GV (0.67±0.79 ml/min) was more efficient in vitreous removal than 23-gauge HV needle 1 (0.26±0.36 ml/min) (*t*-test: *P* < 0.001) (Fig 4). There were no differences in the comparison of 23-gauge GV to HV needles 2 (0.69±0.55 ml/min-*t*-test: *P* = 0.928), 3 (0.93±0.71 ml/min-*t*-test: *P* = 0.08), 4 (0.80±0.54 ml/min-*t*-test: *P* = 0.360), or 5 (0.92±0.73 ml/min-*t*-test: *P* = 0.101)).

## Discussion

Although vitrectomy instrumentation has significantly evolved in recent years, and the efforts to improve the efficiency and to optimize this surgical procedure in smaller format cutters have increased in the last few years, there are many independent factors that can influence vitreous cutter flow rates, such as the viscosity of the aspirated fluid, port and shaft diameters, infusion, vacuum, duty cycle, and cutting rate [11, 15–17].

Most of the efforts to date to overcome the reduction in the volume flow to have been directed toward reducing the viscosity of the vitreous by increasing the cut rate of vitreous chopping [12–13]. However, there is a mechanical speed limit: the speed of the vitreous cutter blade and the duty cycle. These factors condition the maximum cut rate [1–2, 5, 11, 14]. In addition, there are some limitations related with the mechanical cutters, such as, the turbulences created by the periodic opening and closing of the port. In the past, increasing the cut rate reduced this turbulence somewhat, but any cutter with a periodically closed port will result in this effect, and the advantage of increasing the cut rate appears to be dwindling.

As the cutting action requires the viscous material to be draw into the port past the outer needle before it can be cut, there is a natural limit to how close a surgeon can bring the active cutting region to the retina. Also, the outer needle port must be large enough to permit a reasonable amount of tissue into it to achieve a cut, this combined with the interest in smaller and smaller gauge instruments, results in higher infusion pressures to support water flow through the large port when the cutter is not cutting, and to overcome the higher inner lumen flow resistance.

Additionally, the guillotine cutters rely on maintaining no gap between the moving inner needle and the port edges. Sometimes, the inner lumen can move away, permitting the vitreous material to sit between the two edges rather than getting cut, aspirating uncut or partially cut vitreous through the port, resulting in direct tractions on the vitreous strands.

Moreover, it has been recently reported that even the new generation of vitrector cutters (double edge blade) have not been able to produce smaller fragment neither increase the viscosity reduction of the vitreous as a function of cute rate between 1500 and 12,000 CPM [18].

The HV vitreous removal system was designed to address some of these limitations of the mechanical cutters. The HVs has only a single needle instead of two needles, this means that the port is continuously open, permitting smaller sizes on the ports and larger inner lumen diameters, therefore, providing lower flow resistance and lower infusion pressures. For that reason, contrary to pneumatic cutters, the HVs needles did not exhibit a relatively more gradual, linear decline in flow rate with increasing % US power.

In addition, the tissue disruption takes place right at the front of the front surface of the port, rather than behind it and this disruption mechanism does not rely on the interference between an inner and outer needle. Theoretically, this combined with the changes in the rheological properties of the vitreous by US could result in a reduction in the direct traction on the vitreous strands during vitrectomy [19].

Overall, in this study, water and vitreous flow rates were stable and reliable for the HV prototype (Fig 4). Both vitreous and water flow increased with use of the HV at increasing aspiration and power levels. This was true for both the 23- and 25-gauge HV.

Performance of the 23- and 25-gauge GV was similar to that of the HV for changes in aspiration levels, increasing flow rates with increasing aspiration. However, for both gauge GVs, water flow decreased with increasing CPM. Increased CPM of the GV decreases the amount of time the port is open, decreasing the flow rate for water. In vitreous, the increased CPM cuts the more solid vitreous strands into smaller chunks that are more easily removed, improving flow rate.

These results are similar to the fluidics results of studies of other GVs [4,20], suggesting that the Stellaris PC vitrector was a reasonable model for a GV.

Hypersonic vitrector water and flow rates consistently increased with increases in aspiration, US power, and port diameter. A model for water flow rates with the HV included two multi-parameter cross-terms (one for port area and the square root of vacuum and a second for area * vacuum) was a good fit for the data. The square root of vacuum term was introduced as the port acts much like a small orifice for water flow, and flow is generally proportional to the square of the pressure difference across the orifice.

A vitreous flow rate model including a single multi-parameter cross term for port area, power, and vacuum was sufficient to describe the current data. Adding a term to the vitreous model including the square root of the vacuum, as was added for the water flow rate model, did not improve the fit, so we stayed with the simpler model.

Overall, for both GVs and HVs, flow rates for 23-gauge needles were greater than that of 25-gauge needles. Flow rates of water ranged from 1.1–2.3 times greater for the 23- than the 25-gauge GVs and HVs. Flow rates of vitreous ranged from 1.7–3.9 times greater for the 23- than the 25-gauge GVs and HVs.

S4A and S4B Fig and showed more vitreous flow for port diameters greater than 5.5 mils. 5.5 mil ports were used in the 25 gauge HVs in this study, but the larger ports used on some of the 23 gauge HVs could be placed on 25 gauge HVs, with a corresponding increase in flow. Water flow decreases and vitreous flow increases with increasing cut rate for GVs [4, 9].

This difference in flow rates between water and vitreous for GVs may lead to increasing traction on the vitreous at higher cut rates [9]. This would not be an issue with the HVs since both water and vitreous flow increase with increasing aspiration and power. Note that the port open duty cycle for GVs varies with cut rate, and generally will decrease with increasing cut rate; the duty cycle by cut rate was not measured directly for this study, but the water flow results reflect this change in open duty cycle. In contrast, water flow in the HV was not strongly influenced by power; this is consistent with the constant 100% open port inherent in the HV design.

Overall, the purpose of our study, as the first investigation of a device of this type, has been to demonstrate non-inferiority of HV against GV cutter (Fig 4), defining non-inferiority in the vitreous removal function as “capable of achieving similar vitreous flow rates without inducing water flow rates significantly greater than those of a standard GV device (and therefore introducing significantly greater variation in flow). The Fig 4 shows that "0.007 and 0.009” port HV cutters at 40% and 50% power provide equivalent or better flow averaged across all vacuums than the best GV flow averaged across all vacuums at any cut rate.

HV flow in vitreous depends on vacuum levels, port size and ultrasonic power. Water flow appears to be only slightly affected by needle gauge or wall thickness. This suggests the size of HV needles may be successfully decreased while maintaining efficacy. Vitreous flow through GVs is dependent on needle gauge, as well as vacuum levels and cut rate.

Although ultrasound vitrectomy was introduced by Girard in 1975 as part of an overall pars plana lensectomy approach, using a large bore needle (20 gage / 36 mil ID, OD unspecified), operated at high amplitude (90 um) and 40 kHz, the technology failed to gain acceptance. However, small incision ultrasound technology has advanced significantly in the past 40 years, allowing surgeons to take advantage of the benefits of ultrasound-based retinal surgery. Furthermore, while the use of much lower amplitude and much smaller needle ports in the HV may make it a less effective device for a pars planar lensectomy than Girard’s proposal, it may serve to limit the output of the needle in a favourable manner.

### Limitations

Although our study was extensive, there were certain limitations, including the study media, experimental design and the number of variations explored.

Water flow is a poor proxy for vitreous flow in GV cutters–it can be seen from the data that it decreases as cut rate increases, while vitreous flow increases to flattens in general as cut rate increases; thus, we measured performance with both water and porcine cadaver vitreous. In addition, regarding to the flow experiments, the weight and the time was recorded manually by two investigators instead of using a LabView data acquisition software. This may have been a source of bias during the performance of the experiments. Also, the occlusion events during the study were not assessed. However, It should be point it out that the liquefying zone of the HV is just outside the port (unlike the GV, which cuts the material only after it has been drawn into the port, and may not cut on every cycle). Clogging of the inner needle on GV cutters is a known low-severity transient effect, reversed by reflux. The HV cutter has no such inner needle, and particles that make it through the port are therefore much smaller than the inner lumen, so that it is unlikely to clog in the same manner. There were no specific occlusion events observed during the data collection for either cutter. Variation in vitreous flow from measurement to measurement, which can be observed in the data, may be due in part to occlusion, or may be due entirely to other factors, such as variation in vitreous cutting or disruption effectiveness.

Porcine cadaver vitreous may be an imperfect substitute for live human vitreous. On one hand, it generally comes from younger, healthier pigs; on the other, it may break down quickly after being harvested, and may be adversely affected by mechanical or thermal stresses during harvesting or shipping. Some of the variation from data point to data point will inevitably be due to variations in the quality of the vitreous used for a specific data point. In general, the result of this will be both an increase in the standard deviation within the measurements, and the potential for some variation in the means. In addition, a large number of prototype device samples were used, in order to assess the impact of a broad range of parameters (wall thickness, port geometry, gauge, power, vacuum). As only one flow data point was taken for each combination, lumen clogs or handpiece malfunctions introduced further potential for variation across the data set. Further measurements on a more restricted set of parameter variations, with more data samples for each variation, would be of interest in the future.

In addition, although these first results might lead us to consider the HV as a promising new alternative to the currently available guillotine-based technology for PPV. Further studies are required to analyse thoroughly and consistently the efficacy of this new technology, adding studies on tissue attraction [21] or fluidic perturbation around the different probes [22].

These limitations notwithstanding, the observed trends across the data as a function of the changes (for instance, more flow through larger ports) is consistent with what might be reasonably expected. In summary, flow rates from the HVs are stable and predictable for all needle sizes tested.

Our study has introduced a new technology that is uniquely versatile in its capability. Safety along with efficacy are the two most important parameters for any new therapy, be it a device or drug [1]. The results of the new HV prototype in this pilot study were comparable to those obtained using currently available 25- and 23-gauge GVs. Hypersonic vitrector performance depends on vacuum and port for water and vitreous; and ultrasonic power for vitreous flow; but does not depend on gauge or wall thickness for either fluid. This data was consistent with our informal laboratory experience, facilitating the development of smaller gauge and more effective instruments at some point in the future.

## Supporting information

S1 Fig **(A)** Water flow in a 23 gauge (G) Guillotine Cutter (GC) at different vaccums as a function of cut rate. (**B)** Water flow in a 23-G GC at different cut rates as a function of vacuum. (C). Water flow in a 25-G GC at different vaccums as a function of cut rate. (D). Water flow in a 25-G GC at different cut rates as a function of vacuum.
(TIFF)

S2 Fig **(A)** Vitreous flow in a 23 gauge (G) Guillotine Cutter (GC) at different vaccums as a function of cut rate. (**B)** Vitreous flow in a 23-G GC at different cut rates as a function of vacuum. **(C)** Vitreous flow in a 25-G GC at different vaccums as a function of cut rate. (**D)** Vitreous flow in a 25-G GC at different cut rates as a function of vacuum.
(TIFF)

S3 Fig **(A).** Water flow in a 25 gauge (G) 0.0055” port HV cutter at different powers as a function of vacuum. (**B)** Water flow in a 23-G 0.0055” port HV cutter at different powers as a function of vacuum. (C). Water flow in a 23-G 0.010” port HV cutter at different powers as a function of vacuum. (D). Water flow in a 23-G 0.010” port HV cutter at different vacuums as a function of power.
(TIFF)

S4 Fig **(A)** 23G vitreous flow as a function of vacuum for all port diameters. **(B)** 23 Gage HV vitreous flow as a function of power for different port diameters.
(TIFF)

## References

[1] Fang SY, DeBoer CM, Humayun MS. Performance analysis of new-generation vitreous cutters. Graefes Arch Clin Exp Ophthalmol 2008;246:61–7. doi: 10.1007/s00417-007-0672-8 17876598 10.1007/s00417-007-0672-8

[2] Hubschman JP, Bourges JL, Tsui I, Reddy S, Yu F, Schwartz SD. Effect of cutting phases on flow rate in 20-, 23-, and 25-gauge vitreous cutters. Retina 2009;29:1289–93. doi: 10.1097/IAE.0b013e3181acd3a9 19730161 10.1097/IAE.0b013e3181acd3a9

[3] Oshima Y, Wakabayashi T, Sato T, Ohji M, Tano Y. A 27-gauge instrument system for transconjunctival sutureless microincision vitrectomy surgery. Ophthalmology. 2010;117:93–102.e2. doi: 10.1016/j.ophtha.2009.06.043 19880185 10.1016/j.ophtha.2009.06.043

[4] Diniz B, Ribeiro RM, Fernandes RB, Lue JC, Teixeira AG, Maia M, et al Fluidics in a dual pneumatic ultra high-speed vitreous cutter system. Ophthalmologica. 2013;229:15–20. doi: 10.1159/000343073 23108417 10.1159/000343073 PMC3651881

[5] Ribeiro RM, Teixeira AG, Diniz B, Fernandes RB, Zhong Y, Kerns R, et al Performance analysis of ultrahigh-speed vitreous cutter system. Retina 2013;33:928–32. doi: 10.1097/IAE.0b013e31826f069e 23416511 10.1097/IAE.0b013e31826f069e

[6] Fujii GY, De Juan E Jr, Humayun MS, Chang TS, Pieramici DJ, Barnes A, et al Initial experience using the transconjunctival sutureless vitrectomy system for vitreoretinal surgery. Ophthalmology 2002;109:1814–20. 12359600 10.1016/s0161-6420(02)01119-3

[7] Fujii GY, De Juan E Jr, Humayun MS, Pieramici DJ, Chang TS, Awh C, et al A new 25-gauge instrument system for transconjunctival sutureless vitrectomy surgery. Ophthalmology 2002;109:1807–12; discussion 1813. Erratum in: Ophthalmology 2003;110:9. 12359598 10.1016/s0161-6420(02)01179-x

[8] Ibarra MS, Hermel M, Prenner JL, Hassan TS. Longer-term outcomes of transconjunctival sutureless 25-gauge vitrectomy. Am J Ophthalmol 2005;139:831–6. doi: 10.1016/j.ajo.2004.12.002 15860288 10.1016/j.ajo.2004.12.002

[9] Magalhaes O Jr, Chong L, DeBoer C, Bhadri P, Kerns R, Barnes A, et al Vitreous dynamics: vitreous flow analysis in 20-, 23-, and 25-gauge cutters. Retina 2008;28:236–41. doi: 10.1097/IAE.0b013e318158e9e0 18301028 10.1097/IAE.0b013e318158e9e0

[10] DeBoer C, Fang S, Lima LH, McCormick M, Bhadri P, Kerns R, et al Port geometry and its influence on vitrectomy. Retina 2008;28:1061–7. doi: 10.1097/IAE.0b013e3181840b64 18779711 10.1097/IAE.0b013e3181840b64

[11] Thompson JT. Advantages and limitations of small gauge vitrectomy. Surv Ophthalmol 2011;56:162–72. doi: 10.1016/j.survophthal.2010.08.003 21236459 10.1016/j.survophthal.2010.08.003

[12] Charles S. An engineering approach to vitreoretinal surgery. Retina 2004;24:435–44. 15187667 10.1097/00006982-200406000-00015

[13] Sharif-Kashani P, Nishida K, Pirouz Kavehpour H, Schwartz SD, Hubschman JP. Effect of cut rates on fluidic behavior of chopped vitreous. Retina 2013;33:166–9. doi: 10.1097/IAE.0b013e31825db758 22914683 10.1097/IAE.0b013e31825db758

[14] Rizzo S, Genovesi-Ebert F, Belting C. Comparative study between a standard 25-gauge vitrectomy system and a new ultrahigh-speed 25-gauge system with duty cycle control in the treatment of various vitreoretinal diseases. Retina 2011;31:2007–13. doi: 10.1097/IAE.0b013e318213623a 21685823 10.1097/IAE.0b013e318213623a

[15] Jackson T. Modified sutureless sclerotomies in pars plana vitrectomy. Am J Ophthalmol. 2000; 129(1):116–7. 10653434 10.1016/s0002-9394(99)00377-3

[16] Lima LH, Deboer C, McCormick M, Kerns R, Bhadri P, Humayun MS. A new dual port cutter system for vitrectomy surgery. Retina. 2010;30(9):1515–9. doi: 10.1097/IAE.0b013e3181ea48f9 20924265 10.1097/IAE.0b013e3181ea48f9

[17] Fabian ID, Moisseiev J. Sutureless vitrectomy: evolution and current practices. Br J Ophthalmol 2011;95:318–24. doi: 10.1136/bjo.2009.176495 20733022 10.1136/bjo.2009.176495

[18] Rossi T, Querzoli G, Angelini G, Malvasi C, Rossi A, Morini M, et al Hydraulic Resistance of Vitreous Cutters: The Impact of Blade Design and Cut Rate. Transl Vis Sci Technol. 2016;5(4):1 eCollection 2016 Jul 1. doi: 10.1167/tvst.5.4.1 27441099 10.1167/tvst.5.4.1 PMC4942252

[19] Leitgeb N, Schuy S, Zirm M. Ultrasonic vitrectomy—an alternative technique to presently used mechanical procedures. Experimental results. Albrecht Von Graefes Arch Klin Exp Ophthalmol 1979;209:263–8. 311602 10.1007/BF00419061

[20] Matsuoka N, Teixeira A, Lue JC, Fang S, Kerns R, Bhadri P, et al Performance analysis of millennium vitreous enhancer™ system. Ophthalmic Surg Lasers Imaging 2011;42:162–7. doi: 10.3928/15428877-20101223-03 21210579 10.3928/15428877-20101223-03

[21] Dugel PU, Zhou J, Abulon DJ, Buboltz DC. Tissue attraction associated with 20-gauge, 23-gauge, and enhanced 25-gauge dual-pneumatic vitrectomy probes. Retina. 2012;32(9):1761–6. doi: 10.1097/IAE.0b013e3182456f4d 22466488 10.1097/IAE.0b013e3182456f4d

[22] Rossi T, Querzoli G, Angelini G, Malvasi C, Iossa M, Placentino L, et al Fluid dynamics of vitrectomy probes. Retina. 2014;34(3):558–67. doi: 10.1097/IAE.0b013e3182a0e628 24013257 10.1097/IAE.0b013e3182a0e628

