# Supplementary figures and images for: Performance analysis of a new hypersonic vitrector system
Source: PLoS One. 2017 Jun 6;12(6):e0178462. doi: 10.1371/journal.pone.0178462 (PMC5460816; doi:10.1371/journal.pone.0178462)

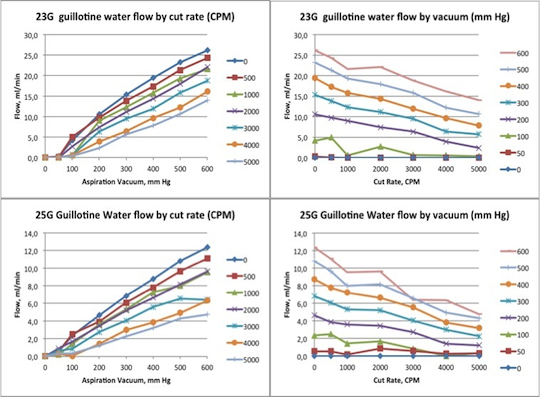

Supplement: S1 Fig — (A) Water flow in a 23 gauge (G) Guillotine Cutter (GC) at different vaccums as a function of cut rate. (B) Water flow in a 23-G GC at different cut rates as a function of vacuum. (C). Water flow in a 25-G GC at different vaccums as a function of cut rate. (D). Water flow in a 25-G GC at different cut rates as a function of vacuum. (TIFF) [file pone.0178462.s001.tiff]

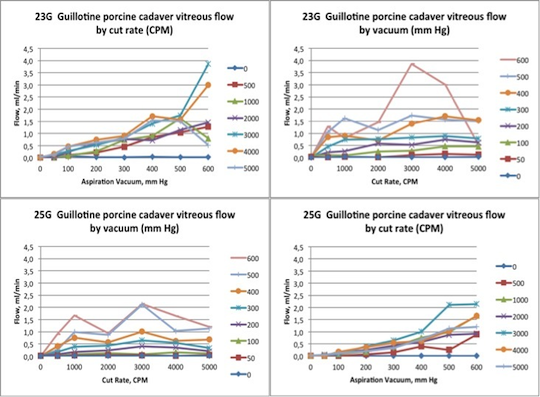

Supplement: S2 Fig — (A) Vitreous flow in a 23 gauge (G) Guillotine Cutter (GC) at different vaccums as a function of cut rate. (B) Vitreous flow in a 23-G GC at different cut rates as a function of vacuum. (C) Vitreous flow in a 25-G GC at different vaccums as a function of cut rate. (D) Vitreous flow in a 25-G GC at different cut rates as a function of vacuum. (TIFF) [file pone.0178462.s002.tiff]

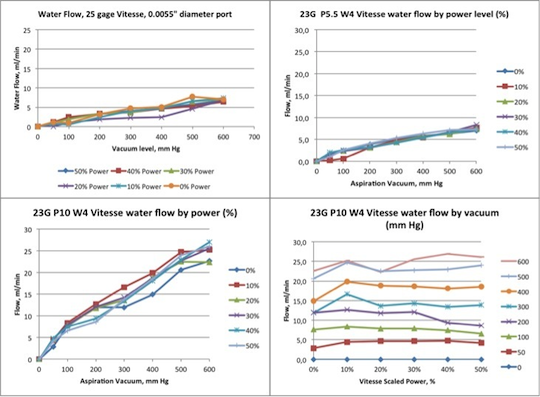

Supplement: S3 Fig — (A). Water flow in a 25 gauge (G) 0.0055” port HV cutter at different powers as a function of vacuum. (B) Water flow in a 23-G 0.0055” port HV cutter at different powers as a function of vacuum. (C). Water flow in a 23-G 0.010” port HV cutter at different powers as a function of vacuum. (D). Water flow in a 23-G 0.010” port HV cutter at different vacuums as a function of power. (TIFF) [file pone.0178462.s003.tiff]

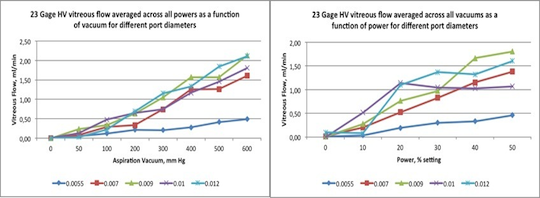

Supplement: S4 Fig — (A) 23G vitreous flow as a function of vacuum for all port diameters. (B) 23 Gage HV vitreous flow as a function of power for different port diameters. (TIFF) [file pone.0178462.s004.tiff]
